# Significant associations between multidimensional factors and physical activity among older adults: an application of Andersen’s behavioral model

**DOI:** 10.3389/fpubh.2026.1756044

**Published:** 2026-01-20

**Authors:** Zisheng Peng, Yibo Zhang, Ruizhi Yang, Zeyu Wang, Jingjing Guo, Meizhen Zhang

**Affiliations:** School of Physical Education and Health Engineering, Taiyuan University of Technology, Taiyuan, Shanxi, China

**Keywords:** Andersen’s behavioral model, built environment, health of older adults, older adults, physical activity

## Abstract

**Objectives:**

Physical activity plays a crucial role in promoting the health of the older adults. However, previous studies examined the influencing factors of physical activity among the older adults from a single-dimensional perspective, failing to adequately account for the interactions between various factors. Consequently, this study applied Andersen’s behavioral model to examine predisposing, enabling, and need factors associated with physical activity characteristics among the older adults.

**Methods:**

Based on Andersen’s behavioral model, this cross-sectional study analyzes factors affecting physical activity among 586 randomly older selected adults (aged ≥60) in Taiyuan via questionnaires. Factors are categorized into predisposing, enabling, and need dimensions, and are analyzed using ordinal and binary logistic regression.

**Results:**

Predisposing, enabling, and need factors were all associated with physical activity levels and insufficient physical activity among older adults. Specifically, gender (OR = 1.893, 95%CI: 1.303–2.751), age (OR = 1.739, 95%CI: 1.034–2.927), pension insurance purchase (OR = 0.312, 95%CI: 0.194–0.501), community facilities convenience score (OR = 1.363, 95%CI: 1.076–1.727), self-rated unhealthy health (OR = 0.375, 95%CI: 0.167–0.845), general health (OR = 0.372, 95%CI: 0.212–0.653), and relatively healthy (OR = 0.472, 95%CI: 0.281–0.793) were identified as significant predictors of physical activity levels. For insufficient physical activity, the influencing factors included gender (OR = 0.470, 95%CI: 0.302–0.732), age (OR = 0.458, 95%CI: 0.250–0.840), pension insurance purchase (OR = 3.944, 95%CI: 2.322–6.698), convenience of community facilities (OR = 0.718, 95%CI: 0.543–0.948), poor self-rated health (OR = 4.555, 95%CI: 1.811–11.456), and fair self-rated health (OR = 2.533, 95%CI: 1.302–4.926).

**Conclusion:**

Older adults’ physical activity levels needed improvement. Among these factors, age-related limitations can hinder participation in activities, while gender differences result in varying preferences and abilities regarding activities. Promoting pension insurance and improving community facilities could boost activity. Self-assessed health status is strongly associated with levels of physical activity. Governments should implement evidence-based health education, tailored incentives, strengthened community support networks, and upgraded age-friendly infrastructure to boost participation. These measures enhance public health and advance active aging through building age-friendly societies.

## 1 Introduction

The global demographic transition toward population aging presents one of the most significant public health challenges of the 21st century. According to the “2021 National Bulletin on the Development Aging Care,” China’s population aged 60 and above reached 26.736 million (18.9% of the total) by the end of 2021. It is projected to surpass 30% by 2034 and exceed 40% by 2055, marking the transition into a super-aged society (1). Chronic diseases are highly prevalent among older adults in China, affecting about 76.3% of this population. These diseases, which are often insidious, chronic, costly, and hard to cure, pose a major threat to health and place a heavy burden on care and healthcare systems (2). Hence, identifying contributing factors is crucial. In this regard, physical inactivity has been established as a key modifiable lifestyle factor strongly linked to the high rate of chronic diseases in aging populations (3). Consequently, promoting physical activity constitutes an important pathway for improving health outcomes among older adults.

Physical activity (PA) has been defined by the WHO as any bodily movement produced by skeletal muscles that results in energy expenditure (4), has played a pivotal role in public health. Previous studies indicate that insufficient PA contributes to 6–10% of the risk of non-communicable diseases and 9% of premature mortality risks, while increasing PA levels is projected to extend life expectancy by approximately 0.68 years (5). Specifically, enhancing PA can effectively reduce Body Mass Index (BMI), mitigate the risk of inflammatory responses and all-cause mortality among older adults (6, 7), and improve cognitive function (8). However, according to a survey conducted by the Centers for Disease Control and Prevention (CDC), only 40% of older adults have achieved the recommended levels of PA (9), highlighting the pressing need to promote PA among this population.

Existing studies have explored the influencing factors of older adults’ physical activity from perspectives such as individual characteristics, sociological factors, or community-built environments (9, 10). However, few studies have comprehensively integrated predisposing, enabling, and need factors within the specific context of Northern Chinese cities. The Andersen’s behavioral model offers a systematic framework for categorizing and integrating various influencing factors, enabling a comprehensive and structured explanation of health behaviors while minimizing the randomness of these factors (11). Previous studies have successfully applied this model to investigate physical exercise and community health service utilization among older adults in China (12). Building upon this foundation, we Building upon this foundation, we utilized the Andersen’s behavioral model to collect data on individual characteristics, incorporating individual characteristics, health status, and community-built environment data. A multilevel modeling analysis is conducted to identify Critical determinants of insufficient PA in older adults, providing both theoretical insights and practical guidance for promoting PA and achieving “active aging.”

## 2 Methods

### 2.1 Study design and participants

Taiyuan, the capital city of Shanxi Province in China, is the province’s political, economic, cultural, and international exchange hub (13). In terms of the degree of aging, the older adult’s population in Taiyuan City constituted 21.91% of the total registered population, which was comparable to moderately aging cities such as Beijing (24.0%), Tianjin (23.43%), Hangzhou (22.16%), Wuhan (20.95%), and Nanjing (20.85%).

**Figure 1:**
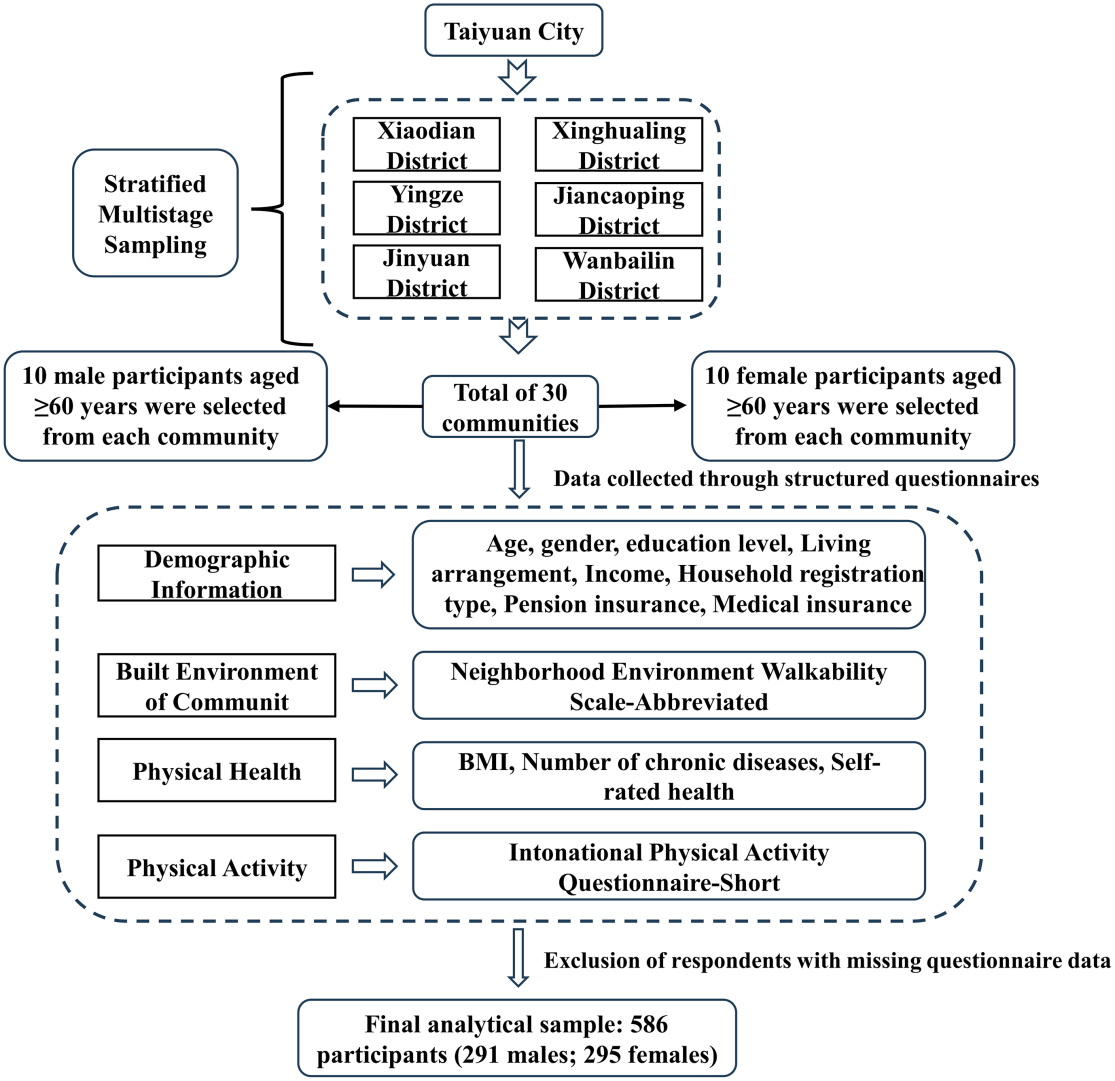
Data collection flowchart.

This was a cross-sectional study conducted from July to August 2024 in Taiyuan, Shanxi Province. A total of 30 communities were selected across six administrative districts (Xiaodian, Yingze, Xinghualing, Jiancaoping, Wanbailin, and Jinyuan) using a multi-level stratified sampling method. Calculation of the sample size required a ratio of sample size to observed variables of 10:1–15:1. The study included 16 variables (eg, demographic variables). To ensure a 20% sample loss rate and the representativeness and accuracy of data, the sample size was calculated to be ≥196 cases (14, 15). Therefore, each community aimed to recruit 20 older adults aged 60 years or older who had resided in the community for at least 6 months and had no cognitive or communication impairments as interviewees, resulting in a planned sample size of 600 respondents. The study primarily collected data on individual characteristics, health status, and the built environment of residential communities. After excluding samples with missing information, a total of 586 valid research samples were obtained, including 291 males and 295 females (Figure 1).

### 2.2 Physical activity of the older adults

The International Physical Activity Questionnaire-Short Form (IPAQ-SF) was used to measure PA of older adults. The reliability and validity of the questionnaire have been confirmed by previous studies (16). The metabolic equivalent (MET) values were set as follows: 8.0 for vigorous-intensity PA, 4.0 for moderate-intensity PA, and 3.3 for walking. The calculation formula was as follows: PA level of a certain intensity per week = MET value of this PA × frequency per week (d/w) × time per day (min/d). The total PA level was calculated as the sum of the three PA levels. Based on the grouping criteria for PA (Table 1), the level of PA was categorized into three tiers: high, medium, and low.

**Table 1:** Physical activity grouping criteria.

Group	Criteria
High	Meet any 1 of the following two criteria: Total high-intensity physical activities ≥3 days/week, with weekly total physical activity level ≥1,500 MET-min/week Combined activities of all three intensities ≥7 days/week, with weekly total physical activity level ≥3,000 MET-min/week
Medium	Meet any 1 of the following three criteria: Engage in ≥3 days/week of high-intensity physical activities (at least 20 min/day) Engage in ≥5 days/week of moderate-intensity activities and/or walking (at least 30 min/day) Combined activities of all three intensities ≥5 days/week, with weekly total physical activity level ≥600 MET-min/week
Low	Meet any 1 of the following two criteria: No reported physical activity Reported some physical activities but did not meet the criteria for Medium or High groups

### 2.3 Individual characteristics and health status of older adults

Drawing on existing research (17), this study collected individual characteristics including gender, age, income, education level, living arrangement, pension insurance, medical insurance, and household registration type via questionnaire survey. An evaluation index for the health status of the older adults was established through three factors: subjective self-rated health, the number of chronic diseases, and BMI, integrating both subjective and objective aspects and encompassing demand factors. Firstly, subjective health was evaluated using a subjective self-rated health scale. Subjective self-rated health demonstrated high consistency with “objective” health evaluations by doctors and was frequently employed as a basis for assessing the health status of the older adults in China (18). This measure was derived from the China Health and Retirement Longitudinal Study (CHARLS) questionnaire, specifically from the question: “How would you rate your health status?” Responses were coded as follows: “Very healthy” = 5 points, “Somewhat healthy” = 4 points, “Neutral “= 3 points, “Somewhat unhealthy” = 2 points, and “Very unhealthy” = 1 point. Scoring followed a positive direction, meaning that a higher score reflects a more positive subjective evaluation of one’s own health (19). Secondly, the number of chronic diseases (hypertension, diabetes, hyperlipidemia) among the older adults was collected via questionnaires. Finally, BMI was calculated by measuring the height and weight of the older adults, applied the formula: BMI = body mass (kg)/height (m)^2^. According to the “The Guidelines for Prevention and Control of Overweight and Obesity of Chinese Adults,” the BMI categories were defined as follows: underweight: BMI < 18.5 kg/m^2^, normal weight: BMI ranging from 18.5 to 24 kg/m^2^, overweight: BMI ranging from 24 to 28 kg/m^2^, and obesity: BMI ≥ 28 kg/m^2^ (20).

### 2.4 Community built environment

For the Community built environment section, we employed the Neighborhood Environment Walkability Scale-Abbreviated (NEWS-A), which has been validated and widely adopted in China as an effective instrument for measuring urban residents’ perceptions of their built environment (21). The measurement content encompassed five dimensions: convenience of supporting facilities, destination accessibility, road connectivity, environmental beautification, and public security. The scale employed a 5-point Likert scale, ranging from strongly disagree (1 point) to strongly agree (5 points), with higher scores reflecting greater satisfaction with the built environment. Cronbach’s *α* was 0.932, indicating high internal consistency of the scale.

### 2.5 Theoretical model

**Figure 2:**
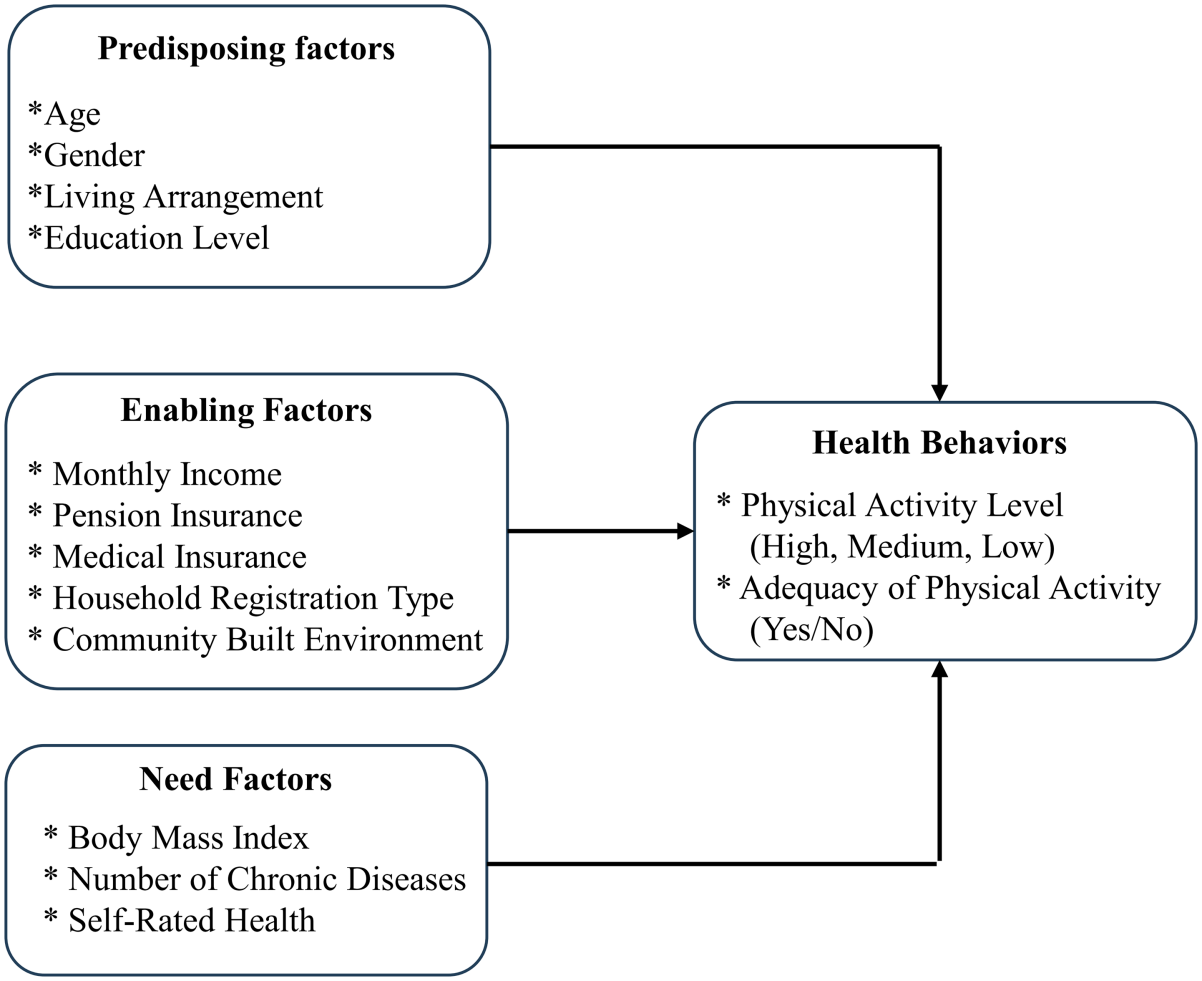
Anderson’s behavioral model.

Based on Andersen’s behavioral model (Figure 2) and drawing upon prior research findings, this study examined the influencing factors of PA among the older adults in Taiyuan from three perspectives: predisposing factors, enabling factors, and demand factors. Detailed variable operationalization and statistical descriptions were presented in Table 2.

(1) Predisposing factors: These primarily encompassed individual characteristics that may influence the PA of the older adults. In this study, demographic characteristics (gender, age) and social structure (education level, living arrangement) were selected as predisposing factors.
(2) Enabling factors: These referred to resource conditions that may affect the PA of the older adults, including personal resources (monthly income, pension insurance, medical insurance, household registration type) and social resources (community-built environment).
(3) Need factors: These involved PA demands potentially generated by individual physical health. In this study, BMI, number of chronic diseases, and self-rated health status were included as need factors.

**Table 2:** Selection and assignment of variables.

Variable classification	Variable	Value description
Predisposing factors	Gender	Male = 1 Female = 0
	Age	60–69 years = 1 70–79 years = 2 ≥80 years = 3
	Education level	No formal education = 0 Primary school = 1 Junior high school = 2 Senior high school/technical school = 3 College or higher = 4
	Living arrangement	Not living with children = 1 Living with children = 2
Enabling factors	Monthly income (CNY)	<¥500 = 1 ¥501–¥1,000 = 2 ¥1,001–¥2,000 = 3 ¥2,001–¥4,000 = 4 >¥4,000 = 5
	Pension insurance	Not enrolled = 0 Enrolled = 1
	Medical insurance	Not enrolled = 0 Enrolled = 1
	Household registration type	Rural = 0 Urban = 1
	Community built environment	Convenience of facilities Accessibility of destinations Road connectivity Esthetic environment Safety and security
Need factors	BMI	Underweight = 1 Normal weight = 2 Overweight = 3 Obese = 4
	Number of chronic diseases	No chronic disease = 3 One chronic disease = 2 Two chronic diseases = 1 Three chronic diseases = 0
	Self-rated health	Very unhealthy = 1 Somewhat unhealthy = 2 Neutral = 3 Somewhat healthy = 4 Very healthy = 5

### 2.6 Statistical analysis

SPSS 26.0 was utilized for data analysis, with frequency and proportion employed to describe the basic characteristics of the older adults population. Based on the Andersen’s behavioral model, ordinal Logistic regression was conducted to explore the influencing factors of PA levels among the older adults, while binary Logistic regression was applied to examine the determinants of insufficient PA in this group. The significance level for all statistical analyses was set at *α* = 0.05.

## 3 Results

### 3.1 Participant characteristics

Table 3 showed a total of 586 older adults individuals aged 60 years and older were included in this survey, composed 291 males and 295 females, with the majority ranged from 60 to 79 years old. Specifically, 72% of the participants held urban household registration, and their educational attainment primarily ranged from primary school to senior high school (or vocational college). Notably, 51.2% of the respondents reported having at least one chronic disease, 41.3% were classified as overweight or obese, and 48.5% rated their health status as “relatively healthy” or better.

**Table 3:** The basic situation of the older adults [*N* (%)].

Category	Variable	Classification	*N* (%)
Predisposing factors	Gender	Male	291 (49.7)
		Female	295 (50.3)
	Age	60–69 years	278 (47.4)
		70–79 years	220 (37.5)
		≥80 years	88 (15.0)
	Education level	No formal education	36 (6.1)
		Primary school	144 (24.6)
		Junior high school	229 (39.1)
		Senior high school/technical school	118 (20.1)
		College or higher	59 (10.1)
	Living arrangement	Not living with children	127 (21.7)
		Living with children	459 (78.3)
Enabling factors	Monthly income (CNY)	<¥500	136 (23.2)
		¥501–¥1,000	40 (6.8)
		¥1,001–¥2,000	47 (8.0)
		¥2,001–¥4,000	200 (34.1)
		>¥4,000	163 (27.8)
	Pension insurance	Not enrolled	125 (21.3)
		Enrolled	461 (78.7)
	Medical insurance	Not enrolled	41 (7.0)
		Enrolled	545 (93.0)
	Household registration type	Rural	164 (28.0)
		Urban	422 (72.0)
Need factors	BMI	Underweight	25 (4.3)
		Normal weight	251 (42.8)
		Overweight	242 (41.3)
		Obese	68 (11.6)
	Number of chronic diseases	Three chronic diseases	54 (9.2)
		Two chronic diseases	58 (9.9)
		One chronic disease	188 (32.1)
		No chronic disease	286 (48.8)
	Self-rated health	Very unhealthy	3 (0.5)
		Somewhat unhealthy	36 (6.1)
		Neutral	181 (30.9)
		Somewhat healthy	284 (48.5)
		Very healthy	82 (14.0)

### 3.2 Multivariate ordinal logistic regression analysis of influencing factors of physical activity level in the older adults

The PA level of older adults was associated with multi-dimensional factors. An ordered logistic regression model was constructed for a comprehensive analysis. Specifically, four models were developed: Model 1 included only predisposing factors as control variables; Model 2 added enabling factors to Model 1; Model 3 incorporated need factors into Model 1; and Model 4 integrated all three types of factors—predisposing, enabling, and need factors. The significance level for hypothesis testing was set at *α* = 0.05. The results were presented as follows:

Model 1: Logit = predisposing factors
Model 2: Logit = predisposing factors + enabling factors
Model 3: Logit = predisposing factors + need factors
Model 4: Logit = predisposing factors + enabling factors + need factors.

Based on the goodness-of-fit test, Model 4 demonstrated the largest *R*^2^ value (Cox and Snell *R*^2^ = 0.146, Nagelkerke *R*^2^ = 0.168), indicating that it provided the strongest explanatory power for the PA level of the older adults. Therefore, Model 4 was selected as the final model to elucidate the influencing factors of PA levels among the older adults population (Table 4).

**Table 4:** Multivariate ordinal logistic regression analysis of factors influencing the level of physical activity in the older adults.

Category	Variable	OR (95%CI)			
		Model 1	Model 2	Model 3	Model 4
Predisposing factors	Gender				
	(ref. male)				
	Female	1.948^*^ (1.416–2.680)	1.960^*^ (1.357–2.831)	1.868^*^ (1.350–2.585)	1.893^*^ (1.303–2.751)
	Age				
	(ref. ≥80 years)				
	60–69 years	1.450 (0.873–2.310)	1.601 (0.955–2.683)	1.454 (0.879–2.408)	1.637 (0.961–2.789)
	70–79 years	1.365 (0.840–2.218)	1.701^*^ (1.024–2.826)	1.396 (0.848–2.300)	1.739^*^ (1.034–2.927)
	Education level				
	(ref. College or higher)				
	No formal education	0.651 (0.291–1.457)	1.151 (0.461–2.876)	0.720 (0.316–1.641)	1.273 (0.500–3.240)
	Primary school	0.645 (0.358–1.160)	0.760 (0.390–1.478)	0.608 (0.334–1.107)	0.715 (0.362–1.414)
	Junior high school	0.754 (0.435–1.307)	0.839 (0.463–1.523)	0.720 (0.412–1.259)	0.799 (0.436–1.466)
	Senior high school/technical school	0.769 (0.421–1.405)	0.831 (0.441–1.566)	0.742 (0.404–1.366)	0.799 (0.421–1.519)
	Living arrangement				
	(ref. Living with children)				
	Not living with children	0.668^*^ (0.448–0.996)	0.721 (0.475–1.094)	0.692 (0.459–1.042)	0.758 (0.494–1.163)
Enabling factors	Monthly income				
	(CNY) (ref. >¥4,000)				
	<¥500		0.755 (0.390–1.462)		0.728 (0.371–1.429)
	¥501–¥1,000		1.649 (0.711–3.826)		1.513 (0.643–3.559)
	¥1,001–¥2,000		1.454 (0.683–3.097)		1.302 (0.601–2.821)
	¥2,001–¥4,000		1.195 (0.753–1.896)		1.252 (0.783–2.001)
	Pension insurance				
	(ref. Enrolled)				
	Not enrolled		0.317^*^ (0.199–0.505)		0.312^*^ (0.194–0.501)
	Medical insurance				
	(ref. Enrolled)				
	Not enrolled		1.329 (0.666–2.651)		1.580 (0.775–3.221)
	Household registration type				
	(ref. Urban)				
	Rural		0.745 (0.448–1.237)		0.771 (0.460–1.291)
	Convenience of facilities		1.356^*^ (1.076–1.728)		1.363^*^ (1.076–1.727)
	Accessibility of destinations		0.979 (0.741–1.294)		1.006 (0.757–1.339)
	Road connectivity		1.135 (0.860–1.498)		1.137 (0.857–1.509)
	Esthetic environment		1.030 (0.808–1.312)		0.994 (0.776–1.273)
	Safety and security		0.892 (0.701–1.134)		0.897 (0.702–1.146)
Need factors	BMI				
	(ref. Obese)				
	Underweight			0.558 (0.221–1.410)	0.532 (0.204–1.388)
	Normal weight			1.130 (0.668–1.913)	1.111 (0.643–1.920)
	Overweight			0.865 (0.514–1.457)	0.849 (0.493–1.464)
	Number of chronic diseases				
	(ref. No chronic disease)				
	Three chronic diseases			1.005 (0.564–1.791)	1.075 (0.584–1.977)
	Two chronic diseases			1.375 (0.782–2.419)	1.355 (0.756–2.429)
	One chronic disease			1.210 (0.840–1.742)	1.092 (0.746–1.599)
	Self-rated health				
	(ref. Very healthy)				
	Very unhealthy			0.183 (0.013–2.510)	0.195 (0.014–2.787)
	Somewhat unhealthy			0.369^*^ (0.169–0.807)	0.375^*^ (0.167–0.845)
	Neutral			0.379^*^ (0.221–0.648)	0.372^*^ (0.212–0.653)
	Somewhat healthy			0.519^*^ (0.316–0.852)	0.472^*^ (0.281–0.793)
Cox and Snell *R*^2^		0.043	0.121	0.073	0.146
Nagelkerke *R*^2^		0.049	0.139	0.084	0.168

^*^Presents a significant difference (*p* < 0.05); All models are consistent with the coding scheme presented in Table 2, with the last category (i.e., the one assigned the largest numerical code) set as the reference group—for example, male for gender and ≥80 years for age.

### 3.3 Multivariate binary logistic regression analysis was used to analyze the influencing factors of insufficient physical activity in the older adults

A further analysis of factors associated with insufficient PA among older adults was conducted. Adequate PA was defined as no less than 75 min of high-intensity exercise or 150 min of moderate-intensity exercise per week, or an equivalent combination of moderate- and vigorous-intensity exercise (with high-intensity exercise time multiplied by 2 equating to moderate-intensity exercise time). Conversely, insufficient PA was defined as failing to meet these criteria (22). Binary Logistic regression analysis was conducted with adequate PA as the reference group. Model 5 was constructed using predisposing factors as control variables, Model 6 incorporated enabling factors based on Model 5, Model 7 included need factors based on Model 5, and finally, Model 8 integrated all three types of factors (predisposing, enabling, and need factors). The significance level was set at *α* = 0.05.

Model 5: Logit = predisposing factors.
Model 6: Logit = predisposing factors + enabling factors.
Model 7: Logit = predisposing factors + need factors.
Model 8: Logit = predisposing factors + enabling factors + need factors.

According to the goodness-of-fit test, Model 8 had the largest *R*^2^ value (Cox and Snell *R*^2^ = 0.172, Nagelkerke *R*^2^ = 0.236), indicating that it provided the highest explanatory power for insufficient PA among the older adults. Therefore, Model 8 was selected as the final model to analyze the influencing factors of insufficient PA in the older adults (Table 5).

**Table 5:** Multivariate logistic regression analysis of factors influencing insufficient physical activity among the older adults.

Category	Variable	OR (95%CI)			
		Model 5	Model 6	Model 7	Model 8
Predisposing factors	Gender				
	(ref. Male)				
	Female	0.486^*^ (0.338–0.699)	0.455^*^ (0.296–0.699)	0.503^*^ (0.346–0.731)	0.470^*^ (0.302–0.732)
	Age (ref. ≥80 years)				
	60–69 years	0.625 (0.369–1.060)	0.503^*^ (0.283–0.894)	0.565^*^ (0.324–0.987)	0.458^*^ (0.250–0.840)
	70–79 years	0.836 (0.496–1.409)	0.647 (0.370–1.131)	0.823 (0.478–1.419)	0.637 (0.356–1.140)
	Education level				
	(ref. College or higher)				
	No formal education	2.849^*^ (1.160–6.997)	1.646 (0.580–4.671)	2.643^*^ (1.040–6.718)	1.551 (0.529–4.545)
	Primary school	1.602 (0.815–3.147)	1.336 (0.615–2.905)	1.595 (0.794–3.204)	1.364 (0.611–3.048)
	Junior high school	1.435 (0.757–2.721)	1.255 (0.620–2.539)	1.522 (0.787–2.943)	1.348 (0.653–2.784)
	Senior high school/technical school	1.226 (0.606–2.480)	1.087 (0.512–2.310)	1.213 (0.589–2.499)	1.089 (0.503–2.361)
	Living arrangement				
	(ref. Living with children)				
	Not living with children	1.598^*^ (1.035–2.468)	1.541 (0.967–2.456)	1.604^*^ (1.019–2.525)	1.564 (0.965–2.536)
Enabling factors	Monthly income				
	(CNY) (ref. >¥4,000)				
	<¥500		1.178 (0.552–2.516)		1.131 (0.515–2.482)
	¥501–¥1,000		0.488 (0.182–1.306)		0.489 (0.178–1.349)
	¥1,001–¥2,000		0.990 (0.412–2.377)		1.019 (0.413–2.514)
	¥2,001–¥4,000		1.056 (0.618–1.803)		1.025 (0.590–1.779)
	Pension insurance				
	(ref. Enrolled)				
	Not enrolled		3.700^*^ (2.223–6.158)		3.944^*^ (2.322–6.698)
	Medical insurance				
	(ref. Enrolled)				
	Not enrolled		0.798 (0.375–1.702)		0.678 (0.306–1.499)
	Household registration type				
	(ref. Urban)				
	Rural		1.617 (0.901–2.900)		1.573 (0.863–2.867)
	Convenience of facilities		0.726^*^ (0.556–0.949)		0.718^*^ (0.543–0.948)
	Accessibility of destinations		0.870 (0.634–1.195)		0.845 (0.608–1.175)
	Road connectivity		0.876 (0.639–1.202)		0.883 (0.639–1.221)
	Esthetic environment		0.850 (0.644–1.121)		0.898 (0.676–1.194)
	Safety and security		1.297 (0.988–1.703)		1.287 (0.971–1.706)
Need factors	BMI				
	(ref. Obese)				
	Underweight			1.616 (0.593–4.402)	1.685 (0.577–4.919)
	Normal weight			0.726 (0.396–1.331)	0.724 (0.379–1.385)
	Overweight			1.036 (0.572–1.875)	1.040 (0.551–1.964)
	Number of chronic diseases				
	(ref. No chronic disease)				
	Three chronic diseases			0.893 (0.474–1.684)	0.779 (0.391–1.551)
	Two chronic diseases			0.513^*^ (0.266–0.989)	0.517 (0.258–1.039)
	One chronic disease			0.617^*^ (0.404–0.941)	0.640 (0.408–1.004)
	Self-rated health				
	(ref. Very healthy)				
	Very unhealthy			3.848 (0.301–49.221)	3.207 (0.234–43.961)
	Somewhat unhealthy			4.574^*^ (1.894–11.046)	4.555^*^ (1.811–11.456)
	Neutral			2.549^*^ (1.359–4.780)	2.533^*^ (1.302–4.926)
	Somewhat healthy			1.647 (0.911–2.975)	1.693 (0.906–3.164)
Cox and Snell *R*^2^		0.051	0.140	0.092	0.172
Nagelkerke *R*^2^		0.070	0.193	0.126	0.236

^*^Presents a significant difference (*p* < 0.05); All models are consistent with the coding scheme presented in Table 2, with the last category (i.e., the one assigned the largest numerical code) set as the reference group—for example, male for gender and ≥80 years for age.

## 4 Discussion

The results indicated that older adults with low, medium, and high levels of PA constituted 41.0, 16.0, and 43.0% of the total population, respectively. Notably, 35.5% of the older adults did not meet the recommended PA guidelines. Therefore, efforts to enhance PA participation among the older adults were warranted.

### 4.1 Association of predisposing factors with physical activity in older adults

In terms of Predisposing factors, demographic characteristics (gender and age) were significantly associated with PA. Specifically, the odds of having a higher PA level were 89.3% higher for women than for men, while their odds of insufficient PA were only 0.47 times those of men. This disparity may have stemmed from differences in physical attributes and traditional gender roles. Chinese women often assumed responsibilities such as caring for the next generation and managing household chores, which facilitated regular low-to-moderate intensity physical activities but left little time for high-intensity exercises (23). In contrast, men generally possessed greater physical fitness, which enabled them to participate more readily in high-intensity activities like lifting heavy objects or engaging in fitness routines, thus increasing their likelihood of engaging in moderate-to-high-intensity physical activities (24). Additionally, PA levels among older adults were closely correlated to age. As age increases, PA levels tended to decline, while the likelihood of insufficient PA rose. For instance, the probability of engaging in PA was 73.9% higher among adults aged 70–79 compared to those aged 80 and above, whereas the probability of insufficient PA among individuals aged 60–69 was only 45.8% that of those aged 80 and above. These findings align with prior research, which attributed the trend to physiological changes associated with aging, including organ degeneration, reduced muscle mass and density, and diminished mobility. Furthermore, concerns about injury and other factors contributed to a gradual reduction in both the duration and intensity of PA as people grew older (25, 26).

### 4.2 Association of enabling factors with physical activity in older adults

The goodness-of-fit test results from the regression analysis indicated that enabling factors showed the strongest association with the PA levels of older adults. Specifically, purchasing pension insurance and subjective evaluations of community support facilities were identified as the primary influencing factors. The findings demonstrated that purchasing pension insurance significantly enhanced PA levels among older adults while reducing the likelihood of physical inactivity. Compared to those who purchased pension insurance, older adults without such insurance exhibited a 68.8% higher probability of lower PA levels and a 294.4% greater likelihood of insufficient PA. This suggested that the financial security provided by pension insurance encourages older adults to engage more actively in outdoor activities and social interactions, thereby improving their PA levels (27). Additionally, this financial security not only enhanced the quality of life for older adults but also fosters a positive attitude and health awareness, motivating them to maintain their wellbeing through active lifestyles (28). However, further analysis revealed no significant association between increased income and PA. Although higher income might increase the frequency of PA, the broader range of recreational options available to high-income older adults could lead to relatively shorter durations and lower intensities of PA (29). In summary, there may be a “bottleneck effect” in the promotion of PA by economic status among older adults. In addition, the findings indicated that greater convenience of community supporting facilities was associated with higher PA levels and a lower probability of physical inactivity among older adults. Specifically, for each point increase in the convenience score of community facilities, the odds of the PA level rising by 36.3% and the odds of insufficient PA decreasing by 28.2%. Previous research corroborated this conclusion, and the convenience of facilities surrounding the residential area could significantly boost the PA of the older adults (17). This was because the fitness service facilities and sports fields accessible in the community offered convenient PA venues and equipment for residents, thereby effectively stimulated the PA behavior of the residents. This convenience encompassed not only the physical accessibility of facilities but also the diversity and quality of facilities, which collectively promoted the PA participation of the older adults (30).

### 4.3 Association of need factors with physical activity in older adults

Regarding demand factors, only self-rated health was significantly associated with PA. This study found that decreases in self-rated health scores among older adults corresponded to reduced PA levels and an increased likelihood of insufficient PA. While many scholars have extensively explored and confirmed the positive effects of PA on health (31), they often overlooked the critical premise that good physical health serves as the foundation for engaging in PA. Older adults with lower self-rated health scores tend to reduce the time and intensity of PA due to their health conditions. This may stem from fears that excessive PA could exacerbate existing health issues or a lack of motivation caused by physical discomfort (32). A study further corroborated this perspective, indicating that older adults were more likely to participate in physical exercise when their physical condition permits, whereas those with poorer health may limit their participation due to safety concerns and family considerations (33). Furthermore, the results showed no statistically significant association between the incidence of chronic diseases and PA among older adults. However, some scholars argued that older adults with chronic diseases are more inclined to engage in physical exercise to improve their health status and manage disease conditions (34). Conversely, some studies suggest that older adults with chronic diseases exhibit lower PA levels and may opt for lighter physical labor, reducing the duration and intensity of exercise to some extent (35). In conclusion, the relationship between the prevalence of chronic diseases and PA among the older adults remains unclear, and future studies should further investigate this association. Additionally, prior research had indicated that overweight and obese individuals are more likely to require PA to reduce their BMI (36). However, in the present study, a large proportion of participants had normal BMI (42.8%) or were overweight (41.3%), while fewer were underweighted (4.3%) or obese (11.6%). This suggests that the BMI distribution was concentrated in the normal and overweight categories, which may account for the lack of a statistically significant effect of BMI on PA levels.

### 4.4 Limitations

Despite its contributions, this study had certain limitations. First, the cross-sectional design restricted our ability to establish causal relationships between variables, as longitudinal data would have been necessary to explore temporal associations. Second, reliance on subjective measures for assessing PA and perceptions of the built environment might have introduced potential biases. Future studies should incorporate objective measures, such as accelerometers for PA assessment and geographic information systems (GIS) for evaluating the built environment, to enhance result reliability.

## 5 Conclusion

To boost PA participation among the older adults, it was vital to consider various factors. Age and gender were associated with participation in physical activity among older adults, through differences in preferences and age-related functional decline. Enabling factors like pension insurance and community infrastructure could enhance activity levels, while economic status may limit this. Self-rated health also affected willingness and ability to participate. To improve health and support active aging, tailored health education and personalized incentives will be needed. Strengthening social support through pension promotion, better eldercare services, family involvement, and accessible community facilities be able to a favorable environment. These actions will aim to increase activity and achieve active aging goals.

## Data Availability

The original data of this article can be obtained from the author under reasonable circumstances.
